# The “Zero-Gap Protocol” for the Rehabilitation of Severe Jaw Atrophy via the Digital Workflow: A Preliminary Clinical Study

**DOI:** 10.3390/dj14060371

**Published:** 2026-06-15

**Authors:** Alberto Gasbarri, Giulia Caporro, Antonio Capogreco, Maurizio D’Amario, Giulia Ciciarelli, Filippo Giovannetti

**Affiliations:** 1 Postgraduate School of Oral Surgery, Department of Life, Health and Environmental Sciences, University of L’Aquila, 67100 L’Aquila, Italy; 2 Department of Life, Health and Environmental Sciences, University of L’Aquila, 67100 L’Aquila, Italy

**Keywords:** subperiosteal implants, maxillary atrophy, bone segmentation, stereolithographic models, immediate loading

## Abstract

**Background:** Severe jaw atrophy (Cawood and Howell Class V–VI) often renders conventional endosseous implantation unfeasible due to the lack of medullary bone and vascularization. This study presents a digital workflow for customized subperiosteal implants designed to eliminate bone segmentation errors and ensure optimal passive fit. **Methods:** Two clinical cases of severe atrophy—a full-arch maxillary rehabilitation and a unilateral partial rehabilitation—were treated using a prosthetic-driven CAD/CAM workflow. Key innovations included densitometric mapping using Hounsfield Units (HU) to identify high-mineralization zones (+1200 to +1800 HU) for strategic screw fixation. Intraoperatively, cobalt–chrome osteoplasty guides and PMMA check-templates were utilized to validate bone segmentation accuracy in vivo and regularize the cortical base. **Results:** The protocol achieved high precision with a monitored alignment deviation of 0.2 mm. At the 2-year follow-up, clinical and radiographic evaluations (CBCT) confirmed the total absence of gaps at the bone–implant interface. No signs of peri-implantitis, osteolysis, or progressive bone loss were observed, and soft tissues remained stable and healthy. **Discussion:** Success was driven by the rigorous management of the bone–implant interface and the use of preparatory surgical devices to bridge the gap between digital planning and surgical reality. The mechanical stability achieved through divergent fixation vectors prevented stress shielding by converting shear forces into compression, stimulating basal bone density according to Wolff’s Law. **Conclusions:** The standardized digital workflow and the use of preparatory surgical devices in this preliminary study showed that complex rehabilitations can be performed with favorable short-term outcomes. While this approach reduces surgical time and biological stress, further prospective studies are required to confirm its clinical predictability and define next-generation subperiosteal implants as a valid alternative for the management of severely atrophic cases.

## 1. Background

The phenomenon of jaw atrophy is not merely a quantitative volumetric reduction but constitutes a profound qualitative and structural skeletal alteration. In severe cases, specifically identified as stage V and VI according to the Cawood and Howell classification, there is a total involution of the alveolar process [1]. This is caused by the loss of physiological functional loading previously transmitted by the dentition to the basal bone. In the maxilla, this catabolic process follows a centripetal resorption pattern, while in the mandible, it follows a centrifugal pattern; together, these result in drastically reduced of bone volume in both the vertical and transverse dimensions. From a histomorphometric perspective, the most critical aspect of this condition is the near-total disappearance of the medullary and trabecular bone components. The remaining basal bone consists almost exclusively of dense, sclerotic cortical bone, characterized by reduced vascularization and significantly lower osteogenic potential compared to cancellous bone [2,3].

In this biological scenario, the placement of conventional endosseous implants is often impracticable or biologically hazardous [4,5]. The lack of vascularized medullary spaces prevents the formation of a stable blood clot—a mandatory prerequisite for angiogenesis and subsequent osseointegration—while the paucity of the residual ridge poses a concrete risk of implant fenestrations or dehiscent sites. Although regenerative and reconstructive techniques remain a valid therapeutic option, they attempt to recreate bone volume upon a poorly vascularized base, often entailing high morbidity, prolonged healing times, and unpredictable outcomes [6,7].

In this context, customized juxta-osseous implantology is emerging as a viable therapeutic alternative for treating severe atrophy, particularly where conventional surgical protocols present an unfavorable prognostic ratio [8,9]. Rather than seeking endosseous anchorage in a substrate lacking the biological advantages of a medullary component, this method leverages the intrinsic mechanical properties of the residual cortical bone to achieve primary stability [2,5,10]. The clinical indication extends beyond extreme volumetric deficits to cases involving the biological failure of previous regenerative maneuvers. In such instances, scarring and bone sclerosis would render further attempts at guided bone regeneration (GBR) or bone grafting highly susceptible to infectious complications or premature resorption [10,11,12,13]. Furthermore, juxta-osseous surgery effectively addresses the needs of patients with systemic or psychological contraindications to major reconstructive surgery, offering a “one-step” definitive solution that minimizes biological latency and facilitates immediate functional loading in many cases [12,13].

The transition from early to current-generation subperiosteal implants has been marked by intense clinical debate, largely due to the high failure rates associated with pioneering analog protocols. However, the failure of traditional subperiosteal implants was not attributable to the biomechanics themselves, but rather to the technical inability to achieve perfect congruence between the framework and the bone surface [2,3]. The long-term success of the implant critically depends on close contact between the Grade V titanium implant surface and the underlying cortical bone; any discrepancy or poor fit promotes the interposition of fibrous connective tissue, compromising stability and predisposing the site to infection [13,14].

In the digital era, CAD/CAM design has overcome the limitations of analog impressions by providing extreme precision, yet it has introduced a new critical challenge: segmentation error [3,15,16]. During the conversion of Cone Beam Computed Tomography (CBCT) images into three-dimensional models, the software must distinguish bone voxels from surrounding tissues. In the presence of dense fibrous soft tissues or a thickened periosteum adhering to the atrophic ridge, algorithms may erroneously interpret these structures as bone. If left uncorrected, this error leads to the production of an oversized implant that fails to adhere correctly to the cortical surface, ultimately leading to clinical failure [13].

Although various CAD/CAM customized subperiosteal implant workflows have been documented in recent literature, a major technical limitation persists regarding bone segmentation accuracy. Standard digital protocols often suffer from discrepancies caused by thin cortical bone layers or soft tissue interference, which can mislead segmentation algorithms and result in ‘voxel fenestration’ or distorted 3D bone models. Consequently, the fabricated framework may lack a true passive fit. The “Zero-Gap Protocol” specifically addresses this gap in current practice by integrating a guided intraoperative validation phase, ensuring that any virtual planning inaccuracies are physically verified and corrected before final implant positioning.

The therapeutic objective of this study is to evaluate a standardized digital-to-surgical workflow—defined as the “Zero-Gap Protocol”—designed to address the technical discrepancies often encountered in customized subperiosteal implantology. While modern CAD/CAM techniques have significantly improved framework precision, the risk of bone segmentation errors and imaging artifacts remains a primary cause of clinical misalignment.

The proposed protocol differentiates itself by incorporating a mandatory intraoperative validation phase to bridge the gap between virtual planning and surgical reality. By utilizing cobalt–chrome osteoplasty guides in conjunction with biocompatible PMMA check-templates, the surgeon can verify the accuracy of bone segmentation and regularize the cortical base in vivo. This systematic approach aims to ensure optimal congruence and passive fit at the bone–implant interface, potentially enhancing primary stability and long-term biological outcomes for patients with severe jaw atrophy where conventional endosseous or regenerative procedures may be contra-indicated.

## 2. Materials and Methods

The protocol represents a standardized digital-to-surgical workflow designed to overcome the traditional limitations of subperiosteal implants by ensuring an unprecedented level of congruence between the framework and the underlying bone surface. This methodology shifts the operative focus from analog impressions to the proactive management of virtual data, utilizing advanced imaging and customized surgical guides to eliminate anatomical incongruities. The entire decision-making and technical process is governed by a prosthetically driven approach, aimed at transforming complex rehabilitations into predictable and repeatable clinical procedures.

**Figure 1:**
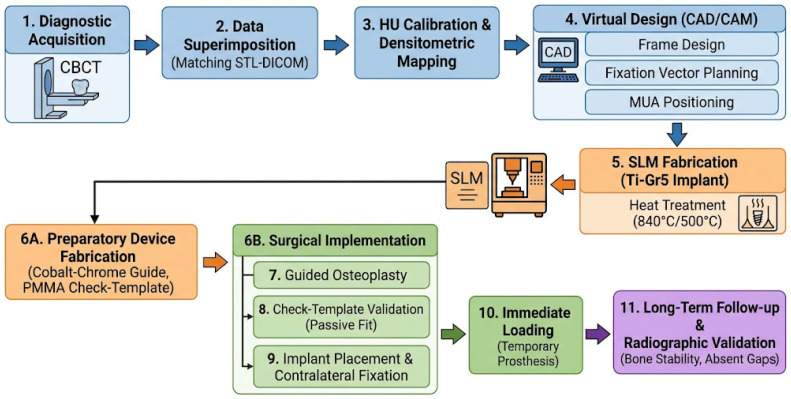
Schematic flowchart of the protocol.

The logical sequence of this protocol is summarized in Figure 1.

### 2.1. Cases Presentation

The following section presents two cases of severe bone atrophy rehabilitated using juxta-osseous implants.

#### 2.1.1. Case 1

A 56-year-old female patient in good general health (ASA II) presented to the authors’ clinic. Her medical history was positive for hypercholesterolemia, pharmacologically managed with atorvastatin.

A thorough clinical and radiographic evaluation was performed. Upon clinical examination, the patient—who wore a removable prosthesis anchored to tooth 2.7 (exhibiting Grade III mobility and periodontal compromise)—reported a history of previous implant failures and severe odontophobia. The patient’s primary request was the restoration of both arches with a fixed dentition.

**Figure 2:**
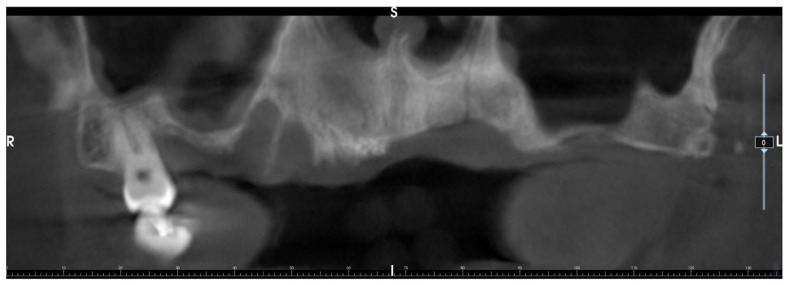
Preoperative orthopantomogram.

The initial orthopantomogram (Figure 2) was followed by a second-level investigation using CBCT, acquired with high-definition parameters: a voxel size of 0.08–0.15, a tube voltage of 90–110 kVp, a tube current calibrated at medium–high mA, and a slice thickness of 0.1–0.3 mm. A wide Field of View (FOV) was utilized: 14 × 14 for the upper jaw and 11 × 11 for the lower jaw.

**Figure 3:**
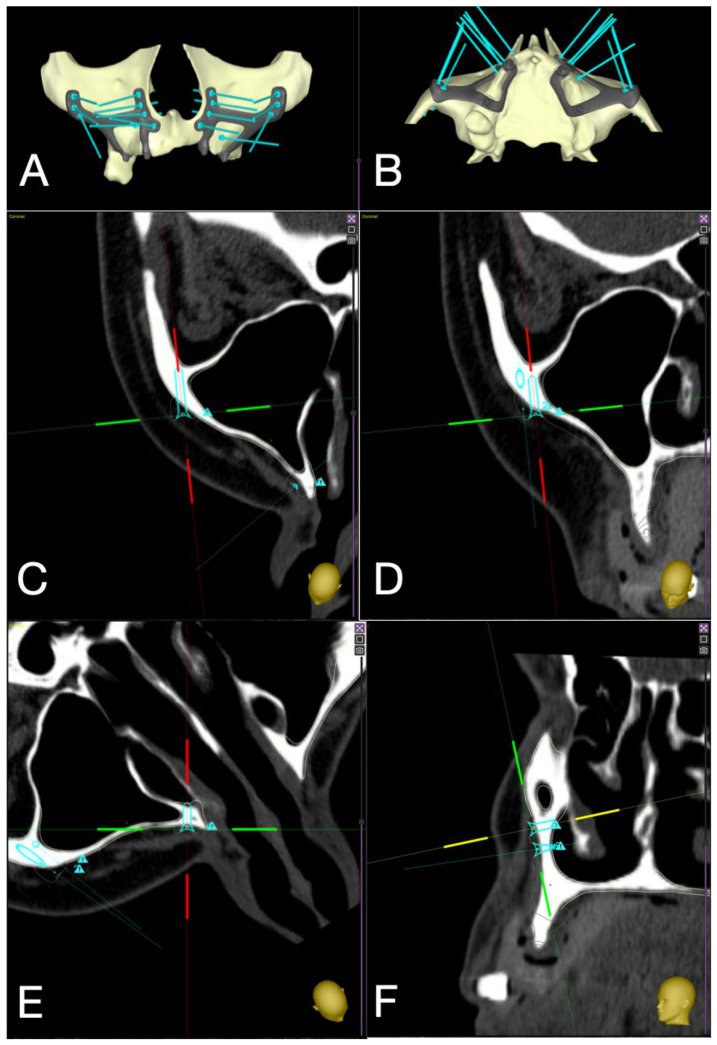
Digital Design and Densitometric Analysis of the Customized Subperiosteal Implant. (**A**,**B**) 3D CAD/CAM modeling of the bilateral subperiosteal frameworks. The design incorporates integrated Multi-Unit Abutments (MUAs) and divergent fixation arms strategically oriented to maximize primary stability through a mechanical “clamp” effect. (**C**,**D**) Coronal Densitometric Mapping of the Zygomatic Buttress. These sections highlight the identification of “high-mineral-density zones” within the zygomatic pillar. The fixation slots are localized in cortical bone areas calibrated between +1200 and +1800 Hounsfield Units (HU), ensuring optimal insertion torque for the synthesis screws. (**E**,**F**) Sagittal Densitometric Mapping of the Canine Pillar. Sections illustrating the engagement of the anterior supporting arms toward the canine pillar, bordering the anterior nasal fossa. The software-monitored alignment confirms a millimetric passive fit with a reported deviation of 0.2 mm, minimizing the risk of “voxel fenestration” and ensuring absolute congruence between the Grade V titanium grid and the atrophic cortical base.

The scan revealed severe maxillary bone atrophy in both the horizontal and vertical dimensions, consistent with Cawood and Howell Class V–VI (Figure 3). Volumetric analysis of the upper arch confirmed insufficient residual alveolar bone in both height and width, rendering the placement of conventional endosseous implants unfeasible without complex and invasive reconstructive bone grafting procedures. Given the patient’s history of implant-prosthetic failures and severe odontophobia, a treatment strategy minimizing invasiveness was preferred. Preference was given to rehabilitation via a customized subperiosteal (juxta-osseous) implant, designed for immediate loading on Multi-Unit Abutments (MUA).

The digital workflow was conducted following a rigorous prosthetically driven approach. In the preliminary phase, the adequacy of the vertical dimension (VD) and aesthetic parameters was verified using the patient’s existing removable prosthesis. Digitalization was achieved via a “double scan” technique. To ensure maximum precision in the alignment of STL and DICOM files, radiopaque markers with a predefined geometric shape recognized by the software were applied to the prosthesis duplicate (radiological template). These markers were present on the STL file, providing a highly accurate reference for data superimposition.

The acquired data were transmitted to the B&B Dental production center (Argelato, Italy) for implant fabrication. To ensure precise reconstruction and minimize scattering, the DICOM dataset was analyzed using B&B Dental GS software (https://bebdental.it/it/pro/soluzioni-digitali/software-chirurgia-guidata/ accessed on 5 June 2026) with HU (Hounsfield Unit) calibration: the maximum value was set to 3000, while the minimum value was calibrated based on the specific bone types of the patient. STL files from the arch scans and prosthetic wax-up were then superimposed onto this base. The 0.2 mm deviation reported was monitored and determined using the proprietary software, which automatically computed the alignment accuracy using a surface-based best-fit registration algorithm targeting the coordinates of the predefined geometric radiopaque markers applied to the radiological template. Subsequently, the modeling of the customized subperiosteal implant was finalized in Meshmixer Version 3.5.474 (https://meshmixer.org), strictly adhering to the surgeon’s clinical specifications.

During the design phase, two distinct juxta-osseous implants were engineered with integrated supporting arms. The positioning of these arms was strategically determined by identifying “high-mineral-density zones” through the calibrated HU values and anatomical analysis of the facial buttresses. The posterior arm extended onto the zygomatic buttress (three fixation slots), while the anterior arm extended toward the canine pillar, bordering the anterior nasal fossa (four slots). This optimized the spatial orientation to leverage the highest mineralization zones for anchorage. To optimize the positioning of prosthetic components within the basal bone, the implants were designed with integrated MUAs to be housed in specific crestal spaces (Figure 3).

The Grade V titanium implant was manufactured using Selective Laser Melting (SLM). To optimize mechanical properties and eliminate internal porosity without volume changes, the framework was heat-treated at 840 °C (for 4 h) and then 500 °C (for 2 h). Millimetric precision of the abutments was ensured by using a five-axis milling machine, and pre-sterilization decontamination was performed using DOWCLENE 1601 (Dow Chemicals Corporation, Midland, MI, USA). The surgical kit was completed with resin anatomical models (Stratasys Objet 30, Stratasys, Eden Prairie, MN, USA), cobalt–chrome osteoplasty guides for bone site preparation, and a biocompatible PMMA check-template.

**Figure 4:**
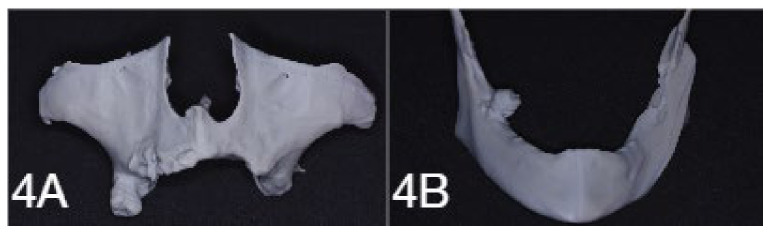
Stereolithographic model. (**A**) Stereolithographic model of the maxilla. (**B**) Stereolithographic model of the mandible.

For final validation, the entire 3D dataset—comprising bone, gingiva, prosthesis, and implants—was re-imported into the B&B Dental GS environment for official surgical approval. A patient-specific stereolithographic model was produced from the CBCT segmentation, allowing the surgeon to optimize preoperative planning and illustrate the atrophic anatomy and surgical dynamics to the patient (Figure 4).

#### 2.1.2. Case 2

A 65-year-old male patient, classified as ASA III, presented with a clinical history of Type II Von Willebrand disease, COPD, and dilated cardiomyopathy, all under strict pharmacological management. The patient required functional and aesthetic restoration via fixed prosthetic rehabilitation, specifically requesting an accelerated treatment protocol.

**Figure 5:**
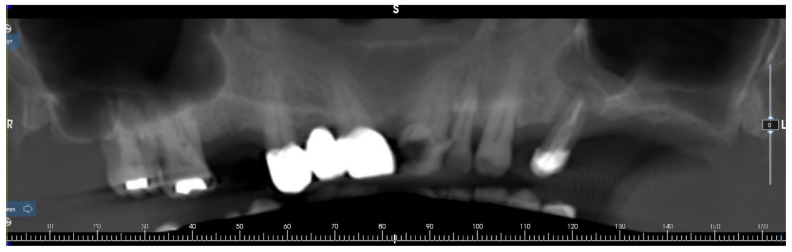
Preoperative orthopantomogram.

A comprehensive clinical and radiographic evaluation was conducted. Physical examination focused on the second quadrant, where tooth 2.5 exhibited advanced periodontitis and Grade III mobility. Initial orthopantomographic screening (Figure 5) followed by second-level CBCT analysis, acquired using high-definition parameters: a voxel size ranging from 0.08 to 0.15 mm^3^, a higher voltage of 90–110 kVp, and medium–high mA. The slice thickness was maintained between 0.1 and 0.3 mm for high-definition detail. A wide Field of View (FOV) was utilized (14 × 14 for the upper jaw and 11 × 11 for the lower jaw) to accurately map the atrophic site.

**Figure 6:**
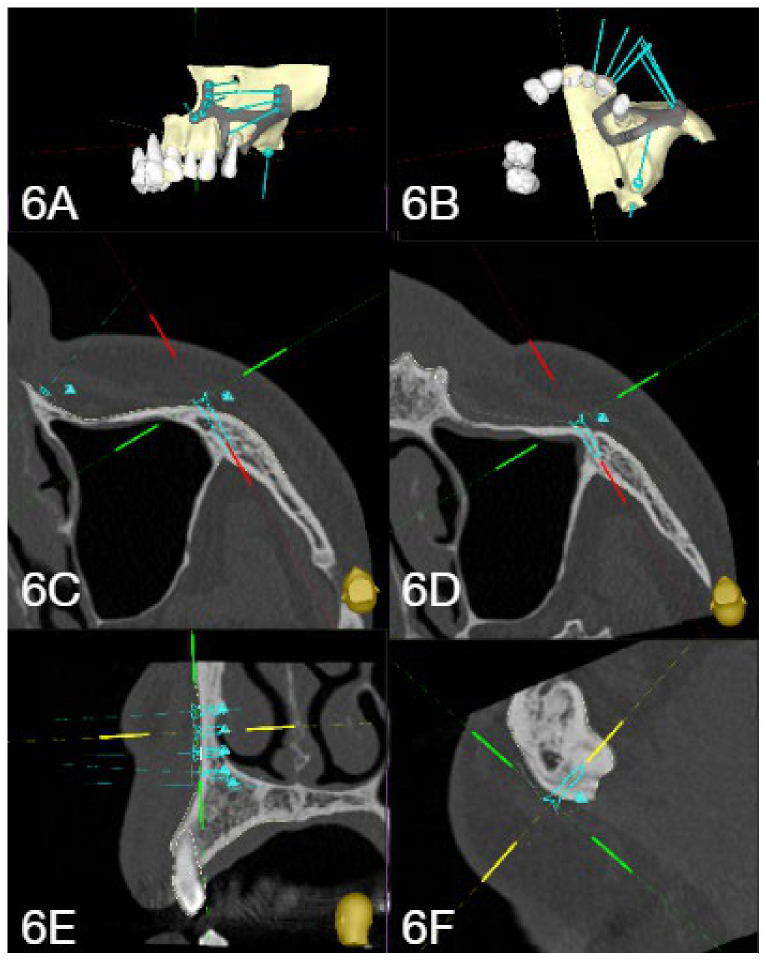
Digital planning and densitometric mapping for Case 2 (Unilateral Maxillary Atrophy). (**A**,**B**) Custom-made subperiosteal implant design. (**C**,**D**) Coronal sections of the second quadrant illustrating the identification of “high-mineral-density zones” within the zygomatic buttress. The software interface highlights the precise fit of the titanium framework against the cortical bone, with densitometric values calibrated between +1200 and +1800 HU to ensure optimal screw stability. (**E**,**F**) Sagittal sections showing the strategic orientation of divergent fixation vectors and bicortical screw engagement. The “Zero-Gap” objective is achieved through a rigorous prosthetically driven workflow, maintaining a monitored alignment deviation of 0.2 mm between the STL prosthetic project and the DICOM bone dataset.

The scan revealed severe unilateral bone atrophy in the affected hemiarch, consistent with Cawood and Howell Class V (Figure 6A). The volumetric deficiency of the residual alveolar ridge precluded the placement of conventional endosseous implants without major regenerative procedures. Consequently, a customized partial juxta-osseous implant was opted for to reduce invasiveness and mitigate hemorrhagic risks associated with the patient’s Type II Von Willebrand disease.

The preoperative workflow followed a rigorous prosthetically driven approach. Digitalization and subsequent matching of DICOM and STL files were performed to develop the virtual project (Figure 6). To ensure maximum precision in data superimposition, radiopaque markers with a predefined geometric shape recognized by the software were applied to the radiological template during the scan, providing highly accurate reference coordinates visible in the STL file. After obtaining informed consent, device design and surgical planning were initiated.

To ensure a precise bone reconstruction and minimize artifacts, the DICOM dataset was analyzed with a specific HU (Hounsfield Unit) calibration: a maximum threshold of 3000 HU was set, while the minimum threshold was dynamically adjusted based on the patient’s specific bone density types. The 0.2 mm deviation reported in the alignment was verified using proprietary software, which provides a visual index to confirm the accuracy of the superimposition between the STL and DICOM files.

The customized juxta-osseous implant featured two primary structural arms. The positioning of these arms was strategically determined through an analytical evaluation of “high-mineral-density zones” identified via the calibrated HU values. This included a posterior extension onto the zygomatic buttress (three slots) and an anterior extension reaching the canine pillar (four slots), ensuring optimal anchorage in the dense facial buttresses (Figure 6).

**Figure 7:**
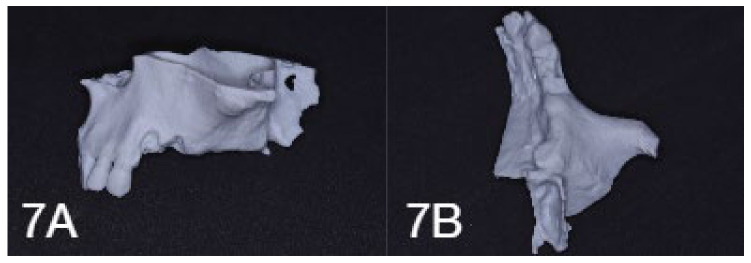
(**A**,**B**) Stereolithographic model.

A stereolithographic model, derived from CBCT bone segmentation, served as both a tactile guide for surgical planning and a visual aid to precisely explain the surgical dynamics to the patient (Figure 7).

## 3. Results

### 3.1. Cases Presentation: Surgical Procedure

#### 3.1.1. Case 1

Due to the patient’s severe odontophobia, the surgery was performed under deep conscious sedation with continuous vital sign monitoring. Local anesthesia was administered via nerve blocks (posterior superior alveolar, greater palatine, nasopalatine, and infraorbital) using an anesthetic solution with 1:100,000 epinephrine. A full-thickness mucoperiosteal flap was reflected from tuberosity to tuberosity, with one midline and two distal releasing incisions. Vestibular and palatal displacement allowed for complete skeletal exposure of the arch, ensuring the identification and protection of the infraorbital nerve, nasal fossae, anterior nasal spine, and incisive canal.

With tooth 1.7 maintained in situ to provide stability, the bone- and tooth-supported osteoplasty guide was positioned and secured with a synthesis screw. The osteotomy was performed using diamond burs, and its accuracy was intraoperatively verified via the check-template. Once the fit was confirmed, tooth 1.7 was extracted.

The surgical phase continued for both the first and second quadrants with the placement of the juxta-osseous implants. After preparing the bone bed, the device was secured using synthesis screws applied in a contralateral sequence to optimize structural stability, tightened to a final torque of 35 Ncm. In the second quadrant, due to poor soft tissue quality and quantity, a pedicled buccal fat pad flap (Bichat’s fat pad) was utilized to increase tissue thickness over the titanium grid. To promote regeneration and increase peri-implant keratinized tissue, a porcine collagen membrane (2 mm thickness, 4–5-month resorption) was placed around the prosthetic connections. The flaps were sutured with interrupted 3/0 Vicryl sutures after applying iodoform paste as an antibacterial treatment.

The procedure concluded with the immediate loading phase. Temporary titanium abutments were placed on the MUAs, onto which a temporary PMMA prosthesis was picked up using dual-cure resin cement. Following extraoral finishing and polishing, the prosthesis was screwed onto the MUA components. Access channels were sealed with composite resin, and occlusion was equilibrated with the antagonist mandibular arch. The postoperative pharmacological regimen included antibiotics, steroids, and analgesics. A postoperative orthopantomogram was then captured.

At the one-week follow-up, the patient reported no complications.

**Figure 8:**
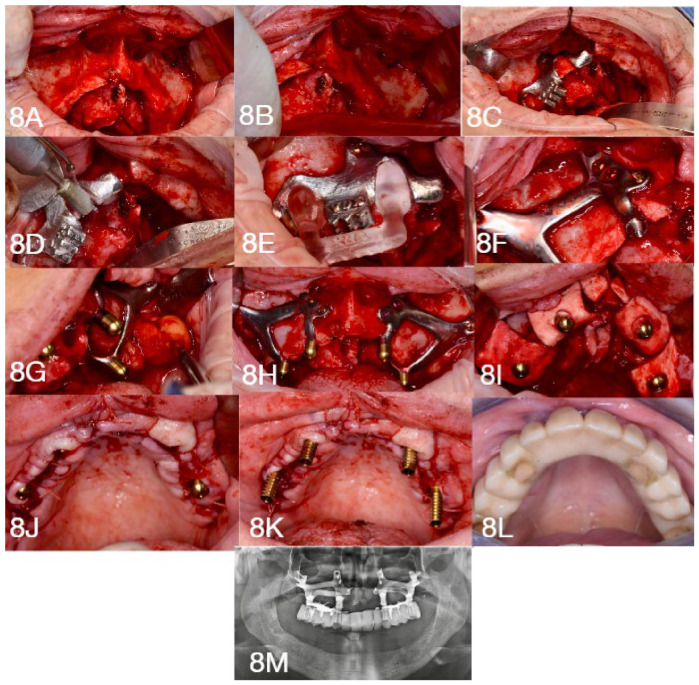
(**A**) Exposure of the surgical site. (**B**) Exposure of the surgical site. (**C**) Placement of the osteoplasty template. (**D**) Osteoplasty using a diamond bur. (**E**) Check-template for site verification. (**F**) Placement of the subperiosteal implant. (**G**) Buccal fat pad (Bichat’s fat pad). (**H**) Placement of the subperiosteal implants. (**I**) Collagen membrane. (**J**) Interrupted sutures. (**K**) Prosthetic phase. (**L**) Prosthetic phase. (**M**) Postoperative orthopantomogram.

The clinical case is presented below in Figure 8.

#### 3.1.2. Case 2

Given the complexity of the patient’s systemic health, the surgery was performed in a protected hospital environment. Due to the patient’s complex medical history, general anesthesia with nasotracheal intubation was selected to secure the airway. This approach allowed for continuous intraoperative monitoring of hemodynamic parameters and coagulation status.

Surgical access was localized to the second quadrant. Following local infiltration of an anesthetic solution with a vasoconstrictor to aid hemostasis, a full-thickness mucoperiosteal flap was reflected via a paramarginal incision extending from the maxillary tuberosity to the canine region, supplemented by distal and mesial releasing incisions. Tissue reflection exposed the residual alveolar ridge and allowed for the identification of critical anatomical structures, including the infraorbital nerve and the zygomatic buttress. The site of tooth 2.5, which had been extracted three weeks prior to surgery to allow for preliminary soft tissue healing, showed satisfactory re-epithelialization.

A bone-supported cutting guide (osteoplasty guide) was positioned on the native bone and stabilized with temporary synthesis screws. Osteoplasty was performed using dedicated rotary instruments to regularize the crest and create a precise housing for the titanium framework. After verifying the congruency of the recipient site with a check-template, the customized partial juxta-osseous implant was inserted and rigidly secured via osteosynthesis screws driven with a final torque of 35 Ncm. The flaps were sutured with interrupted 3/0 Vicryl sutures following the application of iodoform paste as an antibacterial treatment.

The prosthetic phase was optimized through an entirely digital, pre-planned workflow. During the planning stage, the biomedical engineer shared the CAD design files—containing the geometry of the juxta-osseous structure and the spatial coordinates of the prosthetic platforms—with the dental laboratory. This facilitated the pre-surgical fabrication of a provisional PMMA prosthesis, milled based on the virtual anatomy of the approved project.

The prosthesis was delivered and screwed onto the MUA components, and the screw access channels were sealed with composite resin. The procedure concluded with the verification of occlusal balance relative to the antagonist arch. A postoperative orthopantomogram confirmed the correct positioning of the device.

The postoperative pharmacological regimen included antibiotics, corticosteroids, and analgesics. The patient recovered without any postoperative complications.

**Figure 9:**
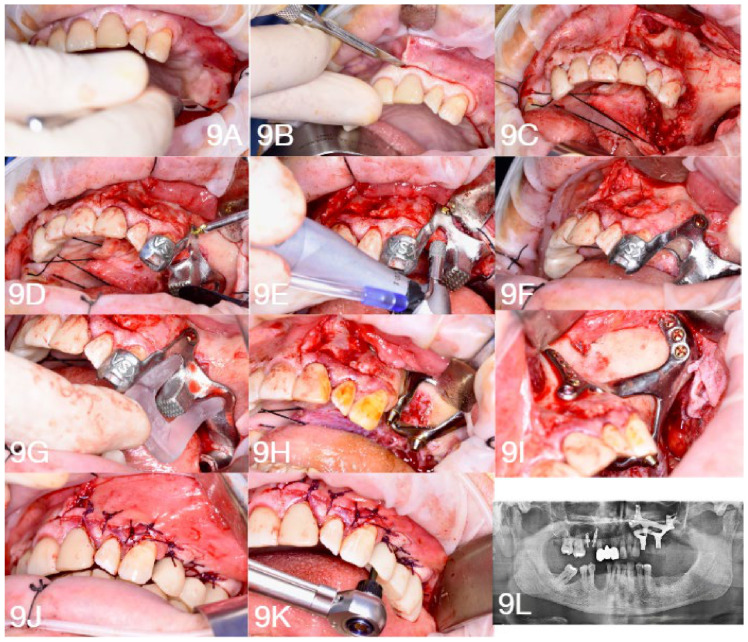
(**A**) Surgical incision. (**B**) Surgical incision. (**C**) Exposure of the surgical site. (**D**) Placement of the osteoplasty template. (**E**) Osteoplasty using a diamond bur. (**F**) Osteotomy. (**G**) Check-template for site verification. (**H**) Placement of the subperiosteal implant. (**I**) Placement of the subperiosteal implants. (**J**) Interrupted sutures. (**K**) Prosthetic phase. (**L**) Postoperative orthopantomogram.

The clinical case is presented below in Figure 9.

### 3.2. Long-Term Clinical and Radiographic Validation of Case 1 and Case 2

**Figure 10:**
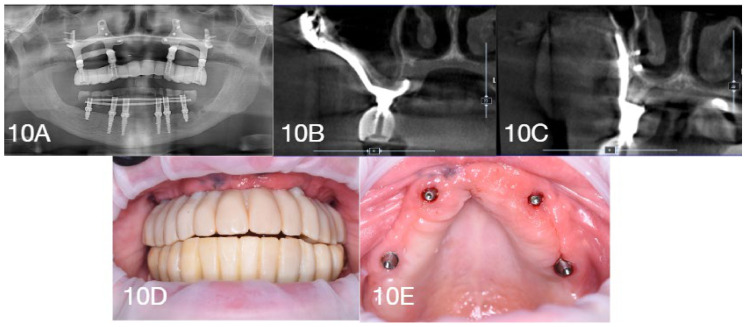
Two-year clinical and radiographic follow-up (Case 1). (**A**) Follow-up orthopantomogram showing the structural integrity of the bilateral full-arch subperiosteal implant. (**B**,**C**) CBCT cross-sections confirming absolute congruence and the absence of gaps at the bone-implant interface. (**D**) Final aesthetic and functional integration at 24 months. (**E**) Intraoral view demonstrating healthy peri-implant mucosa free from signs of inflammation or peri-implantitis.

**Figure 11:**
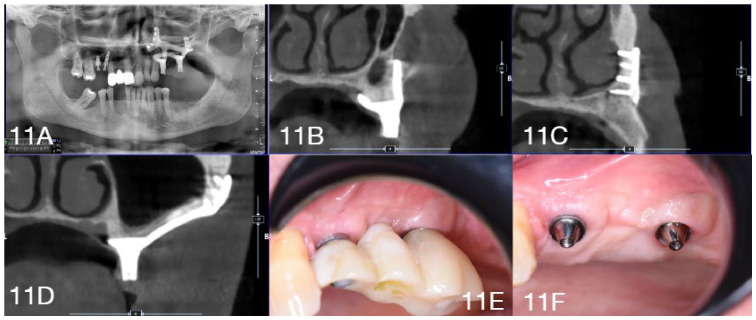
Two-year clinical and radiographic follow-up (Case 2). (**A**) Postoperative orthopantomogram confirming the correct positioning of the partial subperiosteal implant in the second quadrant. (**B**–**D**) High-definition CBCT sections illustrating the stable integration of the titanium grid with the zygomatic buttress and canine pillar, with no visible gaps. (**E**) Functional prosthetic restoration in situ. (**F**) Detail of the MUA platforms showing clinically stable and healthy soft tissues at the 2-year mark.

The implementation of the “Zero-Gap Protocol” facilitated millimetric precision in both clinical cases, with a mean alignment deviation between STL and DICOM datasets consistently monitored at 0.2 mm. The clinical and radiographic evaluation conducted at the 2-year follow-up for both subjects suggests the mid-term technical feasibility and favorable biological response of this standardized workflow. Longitudinal radiographic assessments via orthopantomograms (Figure 10A and Figure 11A) and high-definition CBCT cross-sections (Figure 10B,C and Figure 11B–D) demonstrated remarkable bone stability. Analysis of the bone–implant interface confirmed the complete absence of visible gaps between the Grade V titanium grid and the underlying cortical bone in both the full-arch maxillary rehabilitation (Case 1) and the unilateral partial rehabilitation (Case 2). Furthermore, no gaps were observed between the bone surface and the subperiosteal implant, validating the precision achieved during the digital planning phase.

Radiographically, no signs of peri-implant osteolysis or progressive marginal bone loss were detected around the Multi-Unit Abutment (MUA) platforms in either patient. Clinically, the peri-implant soft tissues appeared highly stable, characterized by healthy, well-keratinized mucosa with no evidence of recession or inflammatory hypertrophy (Figure 10E and Figure 11F). Crucially, no signs of peri-implantitis were observed at either the clinical or radiographic levels; examination confirmed the absence of suppuration, spontaneous bleeding, or bleeding on probing (BoP).

The final prosthetic restorations maintained optimal functional and aesthetic integration (Figure 10D and Figure 11E). In Case 1, three months after the maxillary surgery, the mandibular arch was successfully rehabilitated using an All-on-6 protocol with Monoblock axial implants (Figure 10A), ensuring complete functional restoration for the patient. These findings collectively validate the protocol’s effectiveness in ensuring biomechanical stability according to Wolff’s Law and the long-term biological health of the peri-implant tissues.

### 3.3. Quantitative Metrics and Clinical Outcomes Synthesis

The implementation of the “Zero-Gap Protocol” facilitated millimetric precision across both clinical scenarios, as evidenced by a mean alignment deviation between STL and DICOM datasets consistently maintained at 0.2 mm. This accuracy was underpinned by high-definition diagnostic parameters—utilizing a voxel size of 0.08–0.15 mm^3^ and a slice thickness of 0.1–0.3 mm—which ensured highly faithful bone reconstruction. Furthermore, Hounsfield Unit (HU) calibration (up to 3000 HU) allowed for strategic densitometric mapping, localizing synthesis screws in areas of peak mineralization between +1200 HU and +1800 HU to secure the primary stability required for immediate loading.

From a biological and mechanical perspective, the Grade V titanium frameworks (SLM-manufactured and heat-treated at 840 °C and 500 °C) demonstrated sustained bone-to-implant contact. Longitudinal CBCT assessments at the 2-year follow-up confirmed the total absence of visible gaps at the bone–implant interface, validating the efficacy of the intraoperative osteoplasty and the precision of the digital segmentation. Clinical monitoring throughout the follow-up period revealed optimal peri-implant health, with stable soft tissues and a complete absence of peri-implantitis, suppuration, or progressive bone loss around the Multi-Unit Abutment (MUA) connections.

Regarding Patient-Reported Outcome Measures (PROMs), clinical assessments indicated high patient satisfaction linked to the “one-step” nature of the procedure. This approach effectively bypassed the need for invasive regenerative techniques, significantly reducing biological stress and operative times (ranging from 60 to 120 min). At the final evaluation, both subjects maintained functional and aesthetic integration with no reported complications, confirming the protocol’s reliability in treating “impossible” atrophic cases (Cawood & Howell Class V and VI).

The clinical characteristics, quantitative metrics, and longitudinal outcomes for both cases are synthesized in Table 1.

## 4. Discussion

A critical analysis of the clinical outcomes presented underscores that the success of modern juxta-osseous implantology is not solely attributable to the mechanical properties of the device. Instead, it arises from the rigorous management of the bone–implant interface, facilitated by the accuracy of the digital workflow [4,8,17,18,19]. In this scenario, the transition toward CAD/CAM protocols has not eliminated the need for clinical validation; rather, it has shifted the operative focus from the precision of analog impressions to the proactive management of the uncertainty intrinsic to virtual data [9,13,20,21]. Overcoming the historical limitations of this method lies precisely in the ability to govern the discrepancy between the theoretical model and the real anatomy through guided bone rectification. By systematically eliminating anatomical incongruities and unavoidable imaging artifacts, the surgeon compels the clinical reality to coincide exactly with the design model. Only through such intraoperative validation—facilitated by the synergistic use of the osteoplasty guide and the check-template—is it possible to ensure the absolute passive fit that supports the technical predictability of the subperiosteal implant as a feasible therapeutic solution for severe atrophies [2,4].

Biomechanical stability in Cawood and Howell Class V and VI cases is, in fact, intrinsically linked to the degree of basal stability and the identification of high-mineral-density bone clusters, defined as “noble bone” [1]. The accuracy of this protocol is strictly dependent on the quality of the tomographic acquisition, where the selection of the diagnostic modality represents a decisive phase [21,22]. Although DentalScan (spiral CT) currently remains the procedural standard—owing to its excellent spatial resolution and the capacity for absolute calibration of Hounsfield Units (HU)—the use of CBCT is not excluded, as its accuracy correlates with the cortical bone thickness at the recipient site [8,21,23].

Substantial morphological differences exist between the two maxillary bones: in the maxilla, the cortical bone is typically thinner, making DentalScan indispensable to prevent segmentation errors that would compromise the precision of the subperiosteal grid fit [23,24]. In this region, the thinness of the cortex exponentially increases the risk of “partial volume” artifacts, a phenomenon that can generate virtual gaps (“voxel fenestration”), leading to an underestimation of bone thickness [21,22,25]. Conversely, in the mandible, the thickness and density of the cortex allow for high diagnostic fidelity even via CBCT [23,24].

Furthermore, it must be acknowledged that HU values derived from CBCT datasets in this study were utilized primarily as a qualitative-to-semiquantitative guide for spatial orientation rather than an absolute physical metric, given that CBCT lacks a standardized density baseline compared to Multi-Slice Computed Tomography (MSCT). Because the radiographic datasets were acquired across different diagnostic imaging centers, the specific device-dependent grayscale variations represent an inherent limitation. Consequently, thresholding values were dynamically calibrated based on clinical and anatomical reference areas for each patient to mitigate inter-device variability.

In both scenarios, adopting a protocol with a Large Field of View (FOV, e.g., 10 × 16 cm) is fundamental to map remote anchoring structures, such as the zygomatic pillars, while a reduced slice thickness and isotropic voxels are essential to minimize artifacts [21,25]. To optimize the subsequent software segmentation phase, DICOM data must be exported in a non-compressed multi-file format, preserving spatial metadata without smoothing filters.

Thresholding (binarization) must be based on specific density ranges: highly mineralized cortical bone, ideal for micro-screw stability, ranges between +1200 HU and +1800 HU, while dense alveolar bone is isolated between +500 HU and +1000 HU. It is also crucial to include medullary bone (+200 HU to +400 HU) to prevent the resulting STL model from being hollow or exhibiting artificial fenestrations. The implementation of algorithms such as “Region Growing” and the production of 3D stereolithographic models provide the surgeon with a tactile perception of the atrophic structure [26]. The protocol concludes with intraoperative validation using a Cobalt-Chrome osteoplasty guide and a PMMA check-template, necessary to confirm in vivo segmentation accuracy and ensure primary stability according to the biomechanical principles of Wolff’s Law [27].

A cornerstone of the design phase is the integration of densitometric analysis with the device architecture via B&B Dental GS software. This system allows for the mineral density of bone segments to be decoded and osteosynthesis screw holes to be localized by analytically mapping areas of higher density using Hounsfield Units [28,29]. This mapping directs the grid fixation toward the most mineralized portions of the basal bone in the zygomatic buttress, canine pillar, or mandibular base, ensuring optimal insertion torque and the rigid primary stability that is indispensable for immediate loading [18]. Complementing this digital workflow, the accurate segmentation of DICOM data now allows for the production of 3D stereolithographic models, the role of which is decisive in the preoperative planning of severe atrophy cases. The use of these physical models offers the surgeon irreplaceable tactile perception of the residual anatomy and simultaneously facilitates patient communication, visually illustrating the complexity of the case and the characteristics of the customized device, thereby improving the patient’s understanding of the therapeutic plan and facilitating their informed consent [26]. From a mechanical perspective, stability is further enhanced by divergent screw insertion vectors that create a mechanical “clamp” effect. The geometry of the grid itself is designed to convert shear forces into pure compression distributed along the facial resistance pillars. Finite Element Analysis (FEA) studies, such as those reported in the literature, confirm that this uniform stress distribution prevents the phenomenon of stress shielding [12].

Scientifically, stability is enhanced by divergent screw insertion vectors that create a mechanical “clamp” effect, while the grid geometry converts shear forces into pure compression distributed along the facial pillars of resistance. In accordance with Wolff’s Law, this approach prevents stress shielding and enables the maintenance of basal bone density over time through functional loading [2,30,31].

From a biological and microbiological perspective, achieving millimetric congruence (a mean deviation of 0.2 mm) minimizes the space for bacterial biofilm to grow, preventing the chronic inflammation associated with gaps exceeding 150 microns [8,31]. A critical, and often underestimated, prognostic factor in this context is the management of the soft tissues overlying the titanium structure. Long-term stability depends heavily on maintaining an effective mucosal seal to prevent grid exposure, particularly in cases of severe atrophy where keratinized mucosa is scarce. In such scenarios, the use of advanced flaps or the transposition of the Buccal Fat Pad (BFP) is decisive [19,32]. The vascular supply and volume provided by the BFP not only facilitate faster primary healing but also create a tissue thickness that protects the metal-mucosa interface, significantly reducing the risk of dehiscence [19].

The Grade V titanium structure produced via Selective Laser Melting (SLM) exhibits superior cytocompatibility, promoting macrophage polarization toward the pro-regenerative M2 phenotype and accelerated osteoblastic adhesion due to surface microroughness [20]. The implant created through CAD includes relief zones for the peri-implant biological width and favors the recruitment of mesenchymal stem cells through periosteal repositioning, while the tent-pole effect on soft tissues supports aesthetic volumes and improves the prognosis in terms of hygiene. Finally, the prosthetically driven approach reduces intraoperative time and biological stress—a crucial factor for patients with comorbidities for which rapid execution enhances Patient-Reported Outcome Measures (PROMs) [32].

Finally, the prosthetically driven approach reduces intraoperative time and biological stress—a crucial factor for patients with severe odontophobia and a history of implant failures. By bypassing complex reconstructive grafting, this protocol transforms digital potential into repeatable clinical success, ensuring the long-term functional and psychological stability of the patient [11,19].

### Study Limitations

The findings of this study should be interpreted within the context of several limitations. First, the retrospective nature of this case report and the limited sample size—consisting of two clinical cases—restrict the ability to generalize these outcomes to a broader patient population. Although these cases illustrate the technical feasibility and short-term safety of the ‘Zero-Gap Protocol’, the follow-up period remains limited. Further studies are required to fully document long-term biological sustainability, multi-year bone–implant interface stability, and potential late-stage complications such as material fatigue.

Furthermore, the high degree of precision achieved in these cases was dependent on a specific digital workflow and the use of proprietary software (B&B Dental GS) and hardware, which may not be universally accessible. The clinical success reported here is also intrinsically linked to the surgeon’s experience in managing complex atrophies and the use of preparatory surgical devices, such as the osteoplasty guide and PMMA check-template. Therefore, the predictability of this protocol in a multi-operator environment requires further investigation. Future research should focus on prospective, multicenter clinical trials with larger cohorts and standardized follow-up protocols to fully evaluate the long-term clinical predictability and biological sustainability of customized juxta-osseous implants.

## 5. Conclusions

The present study suggests that the rehabilitation of severe jaw atrophy (Cawood and Howell Class V and VI) via customized juxta-osseous implantology represents a technically feasible approach that may address some biological limitations associated with traditional regenerative techniques. The transition from an empirical-analog approach to an entirely digital workflow aims to standardize a historically complex methodology, offering encouraging technical control and reproducibility.

Clinical evidence suggests successful outcomes in the intraoperative validation of the virtual model. The systematic use of the osteoplasty guide and check-template is necessary to eliminate anatomical discrepancies and radiological artifacts, ensuring the millimetric passive fit indispensable for biomechanical stability and the health of peri-implant tissues. The ability to map bone density in Hounsfield Units and design anchorage based on divergent screws is intended to optimize primary stability, potentially rendering immediate loading technically feasible and compatible with the principles of Wolff’s Law over the evaluated short-term period.

From the patient’s perspective, the prosthetically driven protocol drastically reduces morbidity, operative times, and the number of required interventions. This significantly improves Patient-Reported Outcome Measures (PROMs) and makes the procedure accessible even to systemically fragile or coagulopathic individuals.

In conclusion, while these preliminary results indicate the short-term feasibility and safety of the ‘Zero-Gap Protocol,’ hypotheses regarding its long-term biological sustainability require further validation. This study serves as a preliminary validation of the digital-to-surgical workflow; however, multicenter prospective studies and extended follow-up periods are essential to consolidate statistical data and evaluate the long-term predictability of customized juxta-osseous implants in treating severe atrophic cases. The harmonization of materials engineering, radiological precision, and intraoperative clinical verification now defines the new frontier of advanced implant surgery.

## Figures and Tables

**Table 1:** Clinical Parameters and Comparison of Case 1 and Case 2.

Parameter	Case 1	Case 2
Patient Profile	56-year-old Female, ASA II	65-year-old Male, ASA III
Comorbidities	Hypercholesterolemia, severe odontophobia	Von Willebrand disease, COPD, cardiomyopathy
Atrophy Grade	Cawood & Howell Class V–VI	Cawood & Howell Class V (Unilateral)
Anesthetic Protocol	Deep conscious sedation	General anesthesia (nasotracheal)
Fit Accuracy (Deviation) Screw Insertion Torque	0.2 mm (software-monitored via surface best-fit algorithm) 35 Ncm	0.2 mm (software-monitored via surface best-fit algorithm) 35 Ncm
Bone Mapping (HU)	1200–1800 HU	1200–1800 HU
Operative Time	120 min	60 min
Radiographic Fit (2 Year)	No gaps between grid/bone (CBCT)	No gaps between grid/bone (CBCT)
Peri-implant Health	Stable; no signs of peri-implantitis	Stable; no signs of peri-implantitis
Follow-up Duration	2 years; no complications	2 years; no complications

## Data Availability

The data presented in this study are available on request from the corresponding author due to privacy regulations.
